# To clip or to coil for unruptured intracranial aneurysm? A protocol of randomized controlled trial

**DOI:** 10.1097/MD.0000000000024692

**Published:** 2021-03-19

**Authors:** Xiaoshan Huang, Guang Yan, Zhongzong Qin, Gang Zhu

**Affiliations:** Huizhou Central People's Hospital, Huizhou, Guangdong province, China.

**Keywords:** endovascular coiling, intracranial aneurysm, long term efficacy, protocol, surgical clipping

## Abstract

**Introduction:**

Microsurgical clipping and endovascular coiling are the main methods against unruptured intracranial aneurysm (UIA). The craniotomy of surgical clipping may increase the risk of cerebrospinal fluid leakage and infection, damage the brain tissue, produce excessive stimulation to the nerves and blood vessels around the aneurysm, and cause the corresponding neurological deficit. Endovascular coiling could significantly reduce the mortality and disability rate than surgical clipping technique, which made endovascular coiling to become the first choice for the treatment of UIA. However, the long-term results showed attenuated favorable outcomes of coiling over clipping, so it is still in debate whether to clip or to coil. Therefore, we try to conduct a randomized, controlled, prospective trial to assess the long term safety of endovascular coiling therapy against UIA compared with microsurgical clipping technique.

**Methods:**

Parallel-group randomization (1:1) is generated through the random number generator in Microsoft Excel 2010. In this trial, blinding to patients, physicians, and outcome assessors is not possible. Endovascular coiling or surgical clipping will be performed once for each patient in treatment group or control group, respectively. The mRS, overall mortality rate, disability rate, morbidity rate, and occurrence of a major aneurysm recurrence measured at 6 month and 1 year will be recorded.

**Conclusions:**

The findings will be helpful for the choice of endovascular coiling or surgical clipping by assessing the long term efficacy and safety of both operations against UIA.

**Trial registration:**

OSF Registration number: DOI 10.17605/OSF.IO/QYE9F.

## 1 Introduction

Intracranial aneurysm (IA), also known as cerebral aneurysm, is a localized dilation or ballooning in the cerebral vasculature due to the weakness in the wall of blood vessel.^[1,2]^ The prevalence of unruptured IA (UIA) is 7% in China,^[3]^ and an estimated occurrence of 3.2% worldwide.^[4]^ Most UIAs remain asymptomatic through life, but the ruptured aneurysms causing subarachnoid hemorrhage will lead to severe headache, loss of consciousness, seizure, and even life threat. Many ruptures are fatal, and of the patients who survive, approximately 50% will develop serious complications.^[5]^ Therefore, early surgical intervention is important to reducing mortality and disability.

Microsurgical clipping and endovascular coiling are the main methods against IA.^[6]^ The technique of microsurgical clipping has a long history,^[7]^ and it can open the subarachnoid, release bloody cerebrospinal fluid, and reduce the incidence of cerebral vasospasm.^[8]^ Microsurgical clipping remained to be the preferred operation for its advantages of high aneurysm clipping rate and prevention of rerupture bleeding. However, the craniotomy may increase the risk of cerebrospinal fluid leakage and infection, damage the brain tissue, produce excessive stimulation to the nerves and blood vessels around the aneurysm, and cause the corresponding neurological deficit.

Endovascular coiling therapy emerged in the 1990s with the development of detachable coil system.^[9–11]^ It is a minimally invasive technique without a craniotomy, which could greatly reduce the pain of the patient and makes it easier to be accepted.^[12]^ An 18-year-follow-up study of the International Subarachnoid Aneurysm Trial (ISAT) showed that endovascular coiling could significantly reduce the mortality and disability rate than surgical clipping technique, which made endovascular coiling to become the first choice for the treatment of UIA. ISAT also demonstrated that that the rerupture bleeding rate of endovascular therapy is higher than that of surgical clipping technique,^[13]^ and the long-term results showed attenuated favorable outcomes of coiling over clipping,^[14,15]^ so it is still in debate whether to clip or to coil.^[16]^ Therefore, we try to conduct a randomized, controlled, prospective trial to assess the long term efficacy and safety of endovascular coiling therapy against IA compared with microsurgical clipping technique.

## 2 Methods

### 2.1 Study design

**Figure 1:**
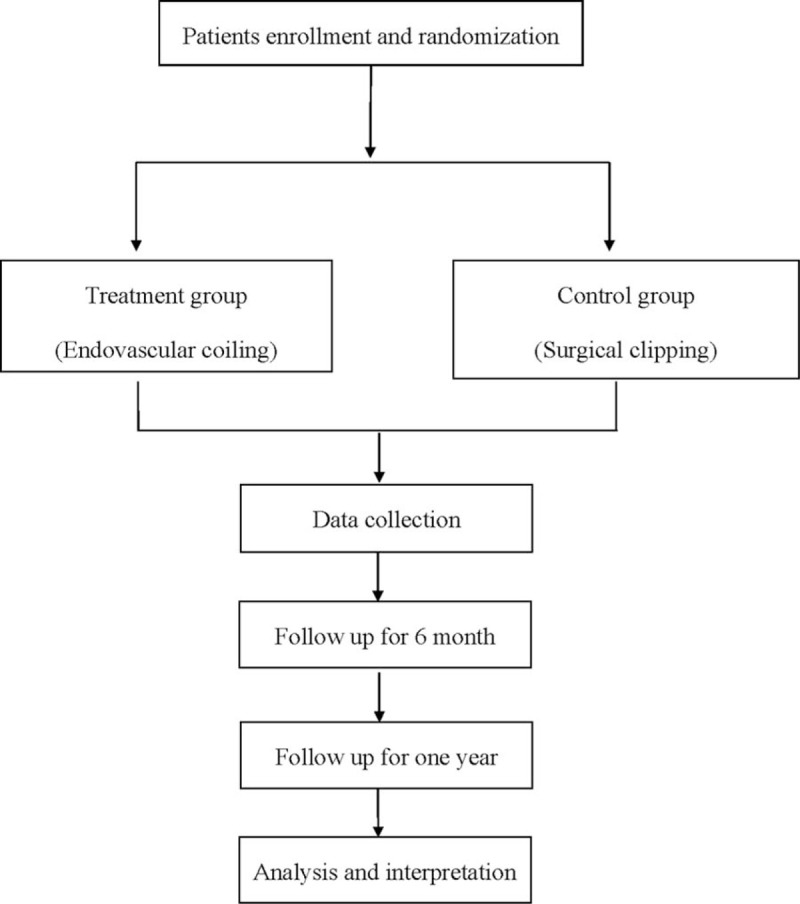
Flow diagram of the study.

The study will be conducted at hospital, and it is a randomized, controlled clinical trial. A flowchart is shown in Figure 1. The study conforms to the SPIRIT 2013 Statement. The protocol will be in accordance with the Helsinki Declaration and approved by the Health Research Ethics Board of our hospital. This experiment has been registered in the open science framework (OSF) (registration number: DOI 10.17605/OSF.IO/QYE9F). Before randomization, all patients will sign a written informed consent and they can freely choose whether to continue the trial at any time.

### 2.2 Participants

Participants’ identification, recruitment, and consecutive enrollment will be all performed in our hospital. Inclusion criteria for participants are: being aged between 18 and 80 years old; having one IA within the anterior circulation, and being considered appropriate for both surgical and endovascular management; exclusion criteria comprise: multiple untreated IAs; an acutely ruptured aneurysm within 14 days of enrollment; modified Rankin Scale (mRS) score of ≥4 or Hunt and Hess score ≥3; cerebral vascular malformation or intracranial mass; a history of ischemic stroke, nervous system tumors, Alzheimer disease, or mental illness.

### 2.3 Randomization and blinding

Parallel-group randomization (1:1) is generated through the random number generator in Microsoft Excel 2010. In this trial, blinding to patients, physicians, and outcome assessors is not possible.

### 2.4 Interventions

Endovascular coiling or surgical clipping will be performed once for each patient in treatment group or control group, respectively.

In the treatment group, the patient is under general anesthesia, and the whole procedure is performed under fluoroscopic imaging guidance. An arterial sheath and angiography catheter are inserted through the femoral artery to assess the shape, size, and location of the aneurysm. An appropriate guiding catheter is inserted and advanced to a site close to the aneurysm. According to the angle of the aneurysm and the artery bearing the aneurysm, a microcatheter is inserted through the initial catheter. When the microcatheter has reached the aneurysm and has been navigated into the aneurysm, select an appropriate coil for embolization according to the actual size of the aneurysm.

In the control group, the patient is under general anesthesia. The appropriate surgical approach is selected based on the location of the aneurysm. Make an arc-shaped incision, separate the subcutaneous tissue, drill the skull, mill the bone window, and expose the aneurysm. Determine the artery bearing the aneurysm, and place a clip on the neck of the aneurysm to close the base of the aneurysm.

### 2.5 Outcome measures

The primary outcomes are mRS, overall mortality rate, disability rate, morbidity rate, occurrence of a major aneurysm recurrence measured at 6 month and 1 year. The secondary outcomes are occurrence of failure of aneurysm occlusion, intracranial hemorrhage during the treatment, length of hospitalization after the treatment. All related complications and adverse events will also be recorded.

### 2.6 Sample size

With alpha 0.05, power 80%, loss to follow-up 20%, and the estimated 18% and 30% of poor clinical outcome rates at 6-month follow-up for endovascular coiling and surgical clipping respectively, a sample size of 450 will be needed.

### 2.7 Statistical methods

The χ^2^ test and Fisher exact test will be applied to categorical variables, and Student *t* test, Wilcoxon rank-sum test, or Mann–Whitney *U* test after normality test for mean values. All analyses will be conducted using SPSS version 22 (IBM, Chicago), and *P* < .05 is considered statistically significant.

## 3 Discussion

Most IAs are clinically silent, and the rupture is unpredictable and associated with hematoma and hemorrhage. With the ongoing advances and widespread use of imaging techniques, the possibility to detect asymptomatic aneurysm has increased significantly.^[17]^ The etiology of IA has not yet been clearly defined, and it is mainly believed to be related to congenital arterial wall defects, increased intravascular pressure, atherosclerosis, and vasculitis.^[18,19]^ Treatment of unruptured IAs is still being discussed. Endovascular therapy has become the main method for the treatment of UIA with the advantages of low fatality and disability rate.^[20–23]^ However, it is still controversial about the choice of endovascular coiling because the long term outcomes are still in debate.^[14,15]^ In order to be helpful for the choice of endovascular coiling or surgical clipping, we try to conduct the study to assess the long term efficacy and safety of both operations against UIA. Additionally, this research includes only patients in 1 hospital in China, and the conclusions will be more limited to Chinese people. And the not applicable blind method will also lead to certain biases for the results.

## Author contributions

**Data collection:** Xiaoshan Huang and Guang Yan.

**Funding support:** Gang Zhu.

**Investigation:** Guang Yan.

**Resources:** Guang Yan and Zhongzong Qin.

**Software operating:** Zhongzong Qin.

**Supervision:** Zhongzong Qin and Gang Zhu.

**Writing – original draft:** Xiaoshan Huang and Guang Yan.

**Writing – review and editing:** Xiaoshan Huang and Gang Zhu.
